# Effect of Honokiol on Cytochrome P450 and UDP-Glucuronosyltransferase Enzyme Activities in Human Liver Microsomes

**DOI:** 10.3390/molecules180910681

**Published:** 2013-09-03

**Authors:** Hyeon-Uk Jeong, Tae Yeon Kong, Soon Sang Kwon, Sung-Woon Hong, Sung Hum Yeon, Jun-Ho Choi, Jae Young Lee, Yong Yeon Cho, Hye Suk Lee

**Affiliations:** 1 College of Pharmacy, the Catholic University of Korea, Bucheon 420-743, Korea; E-Mails: wjd1375@hanmail.net (H.-U.J.); kongtaeyun@naver.com (T.Y.K.); zuzutnseo@naver.com (S.S.K.); yongyeon@catholic.ac.kr (Y.Y.C.); 2 Huons Co., Ltd., Ansan 426-791, Korea; E-Mails: swhong@huons.com (S.-W.H.); yon3547@huons.com (S.H.Y.); cool841123@huons.com (J.-H.C.); leonrekaivalya@huons.com (J.Y.L.)

**Keywords:** honokiol, cytochrome P450 inhibition, UDP-glucuronosyltransferase inhibition, human liver microsomes, drug-drug interaction

## Abstract

Honokiol is a bioactive component isolated from the medicinal herbs *Magnolia officinalis* and *Magnolia grandiflora* that has antioxidative, anti-inflammatory, antithrombotic, and antitumor activities. The inhibitory potentials of honokiol on eight major human cytochrome P450 (CYP) enzymes 1A2, 2A6, 2B6, 2C8, 2C9, 2C19, 2D6, and 3A4, and four UDP-glucuronosyltransferases (UGTs) 1A1, 1A4, 1A9, and 2B7 in human liver microsomes were investigated using liquid chromatography-tandem mass spectrometry. Honokiol strongly inhibited CYP1A2-mediated phenacetin *O*-deethylation, CYP2C8-mediated amodiaquine *N*-deethylation, CYP2C9-mediated diclofenac 4-hydroxylation, CYP2C19-mediated [*S*]-mephenytoin 4-hydroxylation, and UGT1A9-mediated propofol glucuronidation with *K*_i_ values of 1.2, 4.9, 0.54, 0.57, and 0.3 μM, respectively. Honokiol also moderately inhibited CYP2B6-mediated bupropion hydroxylation and CYP2D6-mediated bufuralol 1'-hydroxylation with *K*_i_ values of 17.5 and 12.0 μM, respectively. These *in vitro* results indicate that honokiol has the potential to cause pharmacokinetic drug interactions with other co-administered drugs metabolized by CYP1A2, CYP2C8, CYP2C9, CYP2C19, and UGT1A9.

## 1. Introduction

**Figure 1:**
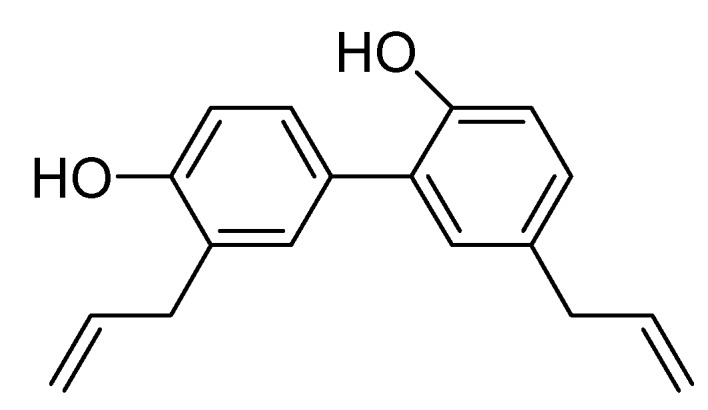
Chemical structure of honokiol.

Honokiol, also known as (2-(4-hydroxy-3-prop-2-enyl-phenyl)-4-prop-2-enyl-phenol, Figure 1), is a biologically active component with antioxidative [1,2,3], anti-inflammatory [4,5,6,7,8], antithrombotic [9], neuroprotective [10,11], antinociceptive [12,13], antidepressant-like [14], and antitumor [15,16,17,18,19,20,21] activities isolated from *Magnolia officinalis*, *Magnolia grandiflora* and other plants. Botanical drugs are widely used by global populations for the prevention and treatment of common illnesses [22]. However, many herb-drug interactions resulting from concurrent use of herbal drugs with prescription and over-the-counter drugs may cause adverse reactions such as toxicity and treatment failure [23,24]. The underlying mechanisms of herb-drug interactions typically involve inhibition or induction of cytochrome P450 (CYP) enzymes, UDP-glucuronosyltransferase (UGT) enzymes, and drug transporters [25,26,27,28,29]. Specifically, St. John’s wort (*Hypericum* *perforatum*), ginkgo (*Ginkgo biloba*), ginseng (*Panax* *ginseng*), milk thistle (*Silybum* *marianum*), and licolice (*Glycyrrhiza* *glabra*) have all been reported to interact with anticoagulants, antiretroviral drugs, anticancer drugs, immunosuppressants, or antidepressants [30,31,32,33]. In addition, bergamotin, a major furanocoumarin found in grapefruit juice, has been reported to increase the blood concentration of drugs by inhibiting hepatic CYP3A activity, thereby enhancing the toxicity of drugs such as simvastatin, felodipine, and cyclosporin [34,35,36]. Therefore, it is necessary to evaluate herb-drug interactions early in order to prevent potentially dangerous clinical outcomes.

To the best of our knowledge, there have been no previous studies that have evaluated the inhibitory effects of honokiol on human CYP and UGT enzymes. In this study, the effect of honokiol on the activity of eight major human CYPs and four major human UGTs were examined using pooled human liver microsomes to evaluate the possibility of honokiol-drug interactions.

## 2. Results and Discussion

The inhibitory effect of honokiol on eight major human CYP enzymes and four major human UGT enzymes were evaluated using a cocktail of CYP probe substrates and each UGT probe substrate in human liver microsomes, respectively. Honokiol potently inhibited CYP1A2-catalyzed phenacetin *O*-deethylation, CYP2C8-catalyzed amodiaquine *N*-deethylation, CYP2C9-catalyzed diclofenac 4-hydroxylation, and CYP2C19-catalyzed [*S*]-mephenytoin 4-hydroxylation with IC_50_ values of 2.1, 8.9, 4.1, and 2.2 μM, respectively (Table 1). Honokiol moderately inhibited CYP2B6-mediated bupropion hydroxylation and CYP2D6-mediated bufuralol 1'-hydroxylation with IC_50_ values of 13.8 and 14.0 μM, respectively. Honokiol weakly inhibited CYP3A-mediated midazolam 1'-hydroxylation with IC_50_ values of 97.3 μM. This was not an unusual finding, given that obovatol, a biphenyl ether lignan, also exhibits an inhibitory effect towards CYP1A2, CYP2B6, CYP2C8, CYP2C9, and CYP2C19 with IC_50_ values of 4.4, 13.9, 11.1, 3.3, and 0.8 μM, respectively [37]. At 100 μM, honokiol produced negligible inhibition of CYP2A6-mediated coumarin 7-hydroxylation. The inhibitory potencies of honokiol were not significantly affected after a 30 min preincubation with human liver microsomes in the presence of NADPH (Table 1), indicating that honokiol does not inhibit CYPs in a time-dependent manner.

**Table 1:** Effect of honokiol on CYP metabolic activity in pooled human liver microsomes (H161).

CYP activity	CYP	IC_50_ (μM) of honokiol	
		no preincubation	with preincubation *
Phenacetin *O*-deethylation	1A2	2.1	4.7
Coumarin 7-hydroxylation	2A6	No inhibition	No inhibition
Bupropion hydroxylation	2B6	13.8	20.8
Amodiaquine *N*-deethylation	2C8	8.9	15.5
Diclofenac 4-hydroxylation	2C9	4.1	3.9
*S*-Mephenytoin 4'-hydroxylation	2C19	2.2	2.9
Bufuralol 1'-hydroxylation	2D6	14.0	38.1
Midazolam 1'-hydroxylation	3A4	97.3	45.8

* Honokiol was preincubated for 30 min in the presence of NADPH before the addition of substrate. No inhibition: inhibition less than 50% at 100 μM of honokiol. The substrate cocktail concentrations used for the assessment of IC_50_ were as follows: 50 μM phenacetin, 2.5 μM coumarin, 2.5 μM amodiaquine, 10 μM diclofenac, 100 μM [*S*]-mephenytoin, 5.0 μM bufuralol, and 2.5 μM midazolam. Inhibition of CYP2B6 activity was separately evaluated using 50 μM bupropion. The data represent the average of three determinations.

**Figure 2:**
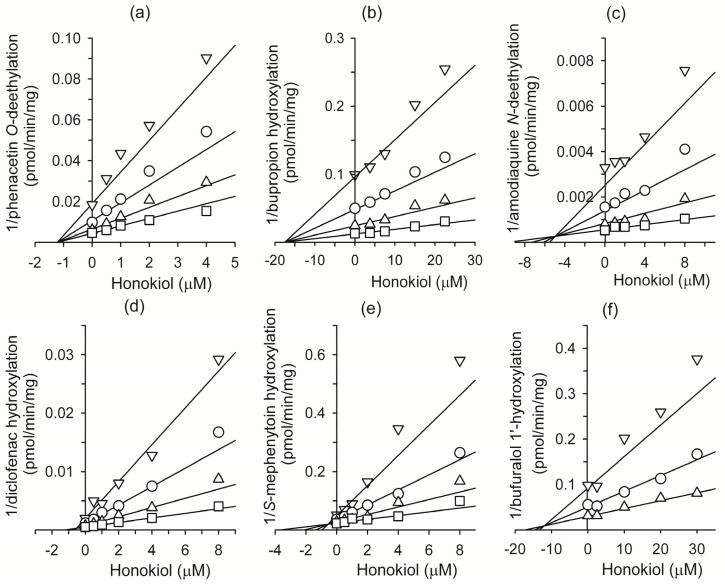
Representative Dixon plots for the inhibitory effects of honokiol on (**a**) CYP1A2-catalyzed phenacetin *O*-deethylation, (**b**) CYP2B6-catalyzed bupropion hydroxylation, (**c**) CYP2C8-catalyd amodiaquine *N*-deethylation, (**d**) CYP2C9-catalyzed diclofenac 4-hydroxylation, (**e**) CYP2C19-catalyzed [*S*]-mephenytoin 4-hydroxylation, and (**f**) CYP2D6-catalyzed bufuralol 1'-hydroxylation in pooled human liver microsomes (H161). Each symbol represents the substrate concentration: (**a**) phenacetin 10 μM (∇), 20 μM (◯), 40 μM (∆), 80 μM (☐), (**b**) bupropion 10 μM (∇), 20 μM (◯), 40 μM (∆), 80 μM (☐), (**c**) amodiaquine, 1.0 μM (∇), 2.0 μM (◯), 4.0 μM (∆), 8.0 μM (☐); (**d**) diclofenac, 1.25 μM (∇), 2.5 μM (◯), 5 μM (∆), 10 μM (☐); (**e**) [*S*]-mephenytoin, 20 μM (∇), 40 μM (◯), 80 μM (∆), 160 μM (☐); (**f**) bufuralol, 1.0 μM (∇), 2.0 μM (◯), 4.0 μM (∆). Each data point represents the mean of triplicate experiments.

In inhibition studies, the apparent *K*_i_ value is a better parameter for defining the interaction of an inhibitor with a particular enzyme. The *K*_i_ values and inhibition types (competitive, noncompetitive, uncompetitive, or mixed) for honokiol were determined using Lineweaver plots, Dixon plots, and secondary reciprocal plots, and the results are summarized in Table 2 and Figure 2. Honokiol noncompetitively inhibited CYP1A2-catalyzed phenacetin *O*-deethylation with a *K*_i_ value of 1.2 μM. Honokiol also competitively inhibited CYP2C9-catalyzed diclofenac 4-hydroxylation (*K*_i_, 0.54 μM), CYP2C19-catalyzed [*S*]-mephenytoin 4'-hydroxylation (*K*_i_, 0.57 μM), CYP2C8-catalyzed amodiaquine *N*-deethylation (*K*_i_, 4.9 μM), CYP2B6-catalyzed bupropion hydroxylation (*K*_i_, 17.5 μM), and CYP2D6-mediated bufuralol 1'-hydroxylation (*K*_i_, 12.0 μM).

**Table 2:** *K*_i_ values for the inhibition of CYP1A2, CYP2B6,CYP2C8, CYP2C9, CYP2C19, CYP2D6, and UGT1A9 activities by honokiol in pooled human liver microsomes (H161).

Enzymes	Marker reactions	*K*_i_ (μM)	Inhibition mode
CYP1A2	Phenacetin *O*-deethylation	1.2	noncompetitive
CYP2B6	Bupropion hydroxylation	17.5	competitive
CYP2C8	Amodiaquine *N*-deethylation	4.9	competitive
CYP2C9	Diclofenac 4-hydroxylation	0.54	competitive
CYP2C19	*S*-Mephenytoin 4'-hydroxylation	0.57	competitive
CYP2D6	Bufuralol 1'-hydroxylation	12.0	competitive
UGT1A9	Propofol glucuronidation	0.3	competitive

**Figure 3:**
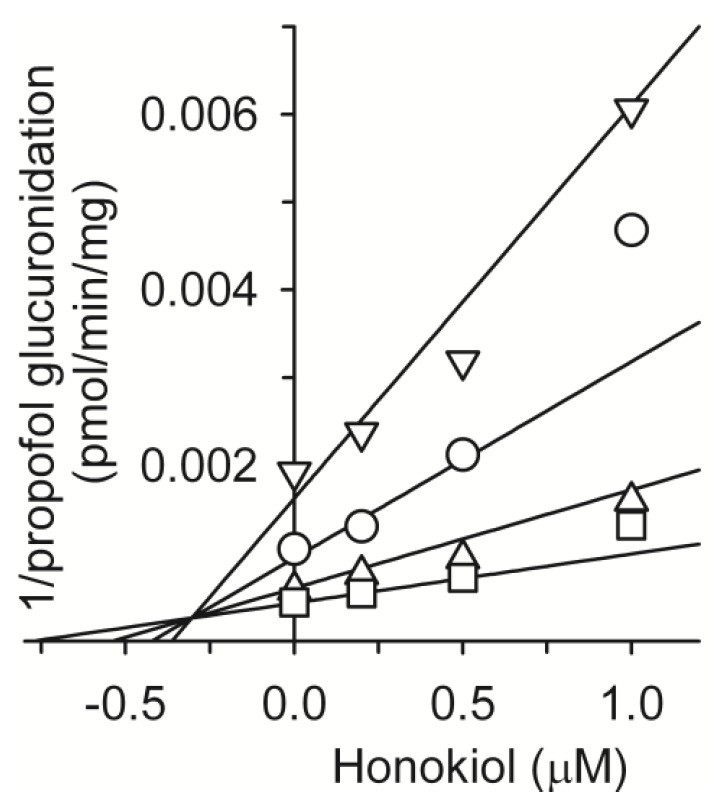
Representative Dixon plots for the inhibitory effects of honokiol on UGT1A9-catalyzed propofolglucuronidation in pooled human liver microsomes (H161). Each symbol represents the substrate concentration: propofol, 5 μM (∇), 10 μM (◯), 20 μM (∆), 40 μM (☐). Each data point represents the mean of triplicate experiments.

We also evaluated the inhibitory potential of honokiol on the activity of four UGT enzymes (Table 3). Honokiol potently inhibited UGT1A9-catalyzed propofol glucuronidation with IC_50_ values of 0.98 μM. Honokiol weakly inhibited UGT1A1-catalyzed 17β-estradiol 3-glucuronidation, UGT2B7-catalyzed azidothymidine glucuronidation, and UGT1A4-catalyzed trifluoperazine *N*-glucuronidation with IC_50_ values of 50.5, 36.4, and 158.1 μM, respectively. Honokiol exhibited competitive inhibition for propofol glucuronidation with a *K*_i_ value of 0.3 μM (Figure 3 and Table 2) and potent propofol glucuronidation inhibitory activity (*K*_i_, 0.3 μM) similar to that of the selective UGT1A9 inhibitor niflumic acid (*K*_i_, 0.1~0.4 μM) [38]. Thus, in order to avoid drug interactions honokiol, it is recommended that it should be used carefully with drugs metabolized by UGT1A9, such as S-etodolac [39], entacapone [40], gaboxadol [41], retigabine [42], and scopoletin [43].

**Table 3:** Effect of honokiol on UGT metabolic activity in pooled human liver microsomes (H161).

UGT	Marker enzyme	IC_50_ (μM)
UGT1A1	17β-estradiol 3-glucuronidation	50.5
UGT1A4	trifluoperazine *N*-glucuronidation	158.1
UGT1A9	propofol glucuronidation	0.96
UGT2B7	azidothymidine glucuronidation	36.4

Honokiol showed potent inhibitory activity against diclofenac hydroxylation (*K*_i_, 0.54 μM) similar to the selective CYP2C9 inhibitor sulfaphenazole (IC_50_ = 0.8 μM) [44], indicating that honokiol should also be used carefully with CYP2C9 substrates such as celecoxib, diclofenac, glyburide, losartan, tolbutamide, torasemide, and *S*-warfarin to avoid drug interactions [45].

Honokiol was shown to be a potent competitive inhibitor of CYP2C19, with a *K*_i_ value of 0.57 μM, indicating that honokiol should be used carefully with CYP2C19 substrates such as diazepam, phenytoin, amitriptyline, imipramine, lansoprazole, and omeprazole in order to avoid drug interactions [46]. In addition, some natural compounds, including anthocyanidin [47], beauvericin [48], corydaline [49], eupatilin [50], and ursolic acid [51] have demonstrated strong inhibition of CYP2C19.

Honokiol was found to be a potent noncompetitive inhibitor of CYP1A2 with a *K*_i_ value of 1.2 μM, indicating that honokiol should be used carefully with drugs metabolized by CYP1A2 such as antipsychotics (clozapine and haloperidol), antiathmatics (theophylline and zileuton), and antidepressants (amitriptyline and clomipramine) in order to avoid drug interactions [46]. Further, several natural compounds including luotonin [52], mollugin [53], and astragaloside IV [54] have been shown to strongly inhibit CYP1A2.

Honokiol was found to be a competitive inhibitor of CYP2C8, with a *K*_i_ value of 4.9 μM, indicating that it should be used carefully with drugs metabolized by CYP2C8 such as cerivastatin, paclitaxel, repaglinide, sorafenib, and torsemide to avoid drug interactions [55]. Its potency was comparable to that of selective CYP2C8 inhibitors such as quercetin (*K*_i_, 2.0 μM) [56], montelukast (*K*_i_, 0.0092–0.15 μM) [57], and gemfibrozil glucuronide (*K*_i_, 20–52 μM) [58].

Herbal preparations containing honokiol may also affect CYP1A2, CYP2C8, CYP2C9, CYP2C19, and UGT1A9 activities. At present, there is no available data on the pharmacokinetics of honokiol in humans, which are indispensable for the prediction of the drug-drug interaction potential of honokiol. These *in vitro* results suggest however that honokiol should be examined for potential pharmacokinetic drug interactions *in vivo* due to its inhibition of CYP1A2, CYP2C8, CYP2C9, CYP2C19, and UGT1A9 activities based on *K*_i_ values of 0.3–4.9 μM.

## 3. Experimental

### 3.1. Materials

Acetaminophen, alamethicin, coumarin, diclofenac, 17β-estradiol, 17β-estradiol 3-glucuronide, glucose-6-phosphate, glucose-6-phosphate dehydrogenase, 7-hydroxycoumarin, midazolam, β-nicotinamide adenine dinucleotide phosphate (NADP), the reduced form of NADP (NADPH), phenacetin, propofol, trifluoperazine, honokiol (>98% by HPLC), and uridine-5-diphospho-glucuronic acid trisoduim salt (UDPGA) were purchased from Sigma-Aldrich (St. Louis, MO, USA). Pooled human liver microsomes (H161), ^13^C_2_, ^15^*N*-acetaminophen, bufuralol, *N*-desethylamodiaquine, 1'-hydroxybufuralol, d_9_-1'-hydroxybufuralol maleate, 4-hydroxy-diclofenac, 4-hydroxymephenytoin, 1'-hydroxymidazolam, and [*S*]-mephenytoin were obtained from BD Gentest Co. (Woburn, MA, USA). Azidothymidine, azidothymidine glucuronide, bupropion, hydroxybupropion, and propofol glucuronide were obtained from Toronto Research Chemicals (Toronto, ON, Canada). Acetonitrile and methanol (HPLC grade) were obtained from Burdick & Jackson Inc. (Muskegon, MI, USA). All other chemicals were of the highest quality available.

### 3.2. Inhibitory Effect of Honokiol on Eight Major CYP Activities in Human Liver Microsomes

The inhibitory potential (IC_50_ values) of honokiol on CYP activities was evaluated in pooled human liver microsomes using liquid chromatography-tandem mass spectrometry (LC-MS/MS). The incubation mixtures were prepared in a total volume of 100 μL as follows: pooled human liver microsomes (0.2 mg/mL), 1.0 mM NADPH, 10 mM MgCl_2_, 50 mM potassium phosphate buffer (pH 7.4), various concentrations of honokiol (0.05–100 μM) and a cocktail mixture of seven CYP probe substrates or bupropion, a CYP2B6-selective substrate, as reported previously [50]. Honokiol was dissolved in acetonitrile. The substrates were used at concentrations approximately equal to or less than that of their respective *K*_m_ values: 50 μM phenacetin for CYP1A2, 2.5 μM coumarin for CYP2A6, 50 μM bupropion for CYP2B6, 2.5 μM amodiaquine for CYP2C8, 10 μM diclofenac for CYP2C9, 100 μM [*S*]-mephenytoin for CYP2C19, 5 μM bufuralol for CYP2D6, and 2.5 μM midazolam for CYP3A4. After a 3 min preincubation at 37 °C, the reactions were initiated by addition of an NADP generating system and incubated for 15 min at 37 °C in a shaking water bath. The reaction was then stopped by placement of the tubes on ice and adding 100 μL of ice-cold methanol containing internal standards (^13^C_2_, ^15^*N*-acetaminophen for acetaminophen and *N*-deethylamodiaquine, and d_9_-1-hydroxybufuralol for 4-hydroxydiclofenac, 4-hydroxybupropion, 7-hydroxycoumarin, 4-hydroxymephenytoin, 1'-hydroxybufuralol, and 1'-hydroxymidazolam). The incubation mixtures were then centrifuged at 13,000 × *g* for 4 min. All incubations were performed in triplicate, and average values were used.

For evaluation of time-dependent inhibition of CYP activities, various concentrations of honokiol (0.05–100 μM) were pre-incubated for 30 min with human liver microsomes in the presence of NADPH. The reaction was initiated by the addition of the cocktail containing seven CYP probe substrates and bupropion.

The metabolites formed from the seven CYP cocktail substrates were simultaneously determined according to our previously described LC-MS/MS method with minor modification [50]; the concentration of 4-hydroxybupropion for CYP2B6 activity was quantified separately by LC-MS/MS. A tandem mass spectrometer (TSQ Quantum Access, Thermo Scientific, San Jose, CA, USA) coupled with a Nanospace SI-2 LC system (Tokyo, Japan) was used. The column and autosampler temperatures were 50 °C and 6 °C, respectively. The mass spectrometer was equipped with an electrospray ionization (ESI) source and operated in positive ion mode. The ESI source settings for ionization of the metabolites were as follows: capillary voltage, 4200 V; vaporizer temperature, 350 °C; capillary temperature 330 °C; sheath gas pressure, 35 psi; auxiliary gas pressure, 15 psi. Quantification was performed by selected reaction monitoring (SRM) of [M+H]^+^ ions and the related product ions for each metabolite. SRM transitions for the metabolites and internal standards have been described previously by our group [50]. Analytical data were processed using Xcalibur^®^ software (Thermo Scientific).

### 3.3. Inhibitory Effect of Honokiol on Four UGT Activities in Human Liver Microsomes

The inhibitory potency (IC_50_ value) of honokiol on UGT1A1-catalyzed 17β-estradiol 3-glucuronidation, UGT1A4-catalyzed trifluoperazine glucuronidation, UGT1A9-catalyzed propofol glucuronidation, and UGT2B7-catalyzed azidothymidine glucuronidation activities was determined in pooled human liver microsomes by LC-MS/MS [48]. Briefly, incubation mixtures were prepared in a total volume of 100 μL as follows: pooled human liver microsomes (0.2 mg/mL for 17 β-estradiol, trifluoperazine, and azidothymidine; 0.1 mg/mL for propofol), 25 μg/mL alamethicin, 10 mM MgCl_2_, 50 mM tris buffer (pH 7.4), each UGT-isoform specific probe substrate (20 μM 17β-estradiol for UGT1A1, 5 μM trifluoperazine for UGT1A4, 10 μM propofol for UGT1A9, or 100 μM azidothymidine for UGT2B7), and various concentrations of honokiol (1–200 μM for UGT1A1, UGT1A4, and UGT2B7; 0.01–2 μM for UGT1A9). Reactions were initiated by the addition of UDPGA (final concentration, 5 mM), and incubations were carried out at 37 °C in a shaking water bath for 30 min. Reactions were terminated by adding 100 μL of ice-cold methanol containing an internal standard (500 ng/mL ezetimibe for 17β-estradiol 3-glucuronide and propofol glucuronide; 30 ng/mL meloxicam for trifluoperazine glucuronide and azidothymidine glucuronide). The incubation mixtures were then centrifuged at 13,000 × *g* for 4 min, followed by dilution of 30 μL of the supernatant with 70 μL of water. An aliquot (5 μL) was injected onto the LC-MS/MS. All incubations were performed in triplicate and the mean values were used. Glucuronides produced from UGT isoform-specific substrates were determined by LC-MS/MS [49].

### 3.4. Kinetic Analysis

In order to determine *K*_i_ values of honokiol for CYP1A2, CYP2B6, CYP2C8, CYP2C9, CYP2C19, and CYP2D6 enzymes, human liver microsomes (0.1 mg/mL for CYP2C8, CYP2C9, 0.15 mg/mL for CYP2B6, and 0.2 mg/mL for CYP1A2, CYP2C19, and CYP2D6) were incubated with various concentrations of substrates (10–80 μM phenacetin for CYP1A2, 10–80 μM bupropion for CYP2B6, 1–8 μM amodiaquine for CYP2C8, 1.25−10 μM diclofenac for CYP2C9, 20–160 μM [*S*]-mephenytoin for CYP2C19, and 1−4 μM bufuralol for CYP2D6, respectively), 1 mM NADPH, 10 mM MgCl_2_, and various concentrations of honokiol in 50 mM potassium phosphate buffer (pH 7.4) in a total incubation volume of 100 μL. The reactions were initiated by the addition of NADPH at 37 °C and stopped after 10 min by placing the incubation tubes on ice and adding 100 μL of ice-cold methanol containing an internal standard (150 ng/mL ^13^C_2_, ^15^*N*-acetaminophen for acetaminophen and *N*-desethylamodiaquine; 10 ng/mL d_9_-1'-hydroxybufuralol for hydroxybupropion, 4-hydroxydiclofenac, 4-hydroxymephenytoin, and 1'-hydroxybufuralol). The incubation mixtures were centrifuged at 13,000 × *g* for 4 min, and the supernatants were diluted as follows: dilution of 20 μL supernatant with 180 μL of 25% methanol for CYP2C8; dilution of 50 μL supernatant with 50 μL of water for CYP2C9, CYP2C19, CYP2D6, and CYP1A2; and dilution of 20 μL supernatant with 80 μL of water for CYP2B6. Aliquots (5 μL) of the diluted supernatants were then analyzed by LC-MS/MS.

For the determination of *K*_i_ values for UGT1A9, human liver microsomes (0.1 mg/mL) were incubated with various concentrations of propofol (5–40 μM), 5 mM UDPGA, 25 μg/mL alamethicin, 10 mM MgCl_2_, and various concentrations of honokiol (0–1 μM) in 50 mM Tris buffer (pH 7.4) in a total incubation volume of 100 μL. The reactions were initiated by addition of UDPGA at 37 °C and stopped after 30 min by placing the incubation tubes on ice and adding 100 μL of 500 ng/mL ezetimibe (internal standard) in ice-cold methanol. The incubation mixtures were then centrifuged at 13,000 × *g* for 4 min, after which 30 μL of the supernatant was diluted with 70 μL of water. Aliquots (5 μL) were then analyzed by LC-MS/MS.

### 3.5. Data Analysis

IC_50_ values (concentration of the inhibitor causing a 50% inhibition of the original enzyme activity) were calculated using Sigma Plot 8.0 (Systat Software, Inc., San Jose, CA, USA). The apparent kinetic parameters for inhibitory potential (*K*_i_ values) were estimated from the fitted curves using Enzyme Kinetics Ver. 1.1 software (Systat Software Inc.).

## 4. Conclusions

The effect of honokiol on eight CYPs and four UGTs was determined across a wide range of substrates and honokiol concentrations using human liver microsomes. CYP1A2, CYP2C8, CYP2C9, CYP2C19, and UGT1A9 activities were potently inhibited by honokiol upon incubation in microsomes. Honokiol weakly inhibited CYP2B6-catalyzed bupropion hydroxylation, CYP2D6-catalyzed bufuralol 1'-hydroxylation, CYP3A-catalyzed midazolam hydroxylation, UGT1A1-catalyzed 17β-estradiol 3-glucuronidation, and UGT2B7-catalyzed azidothymidine glucuronidation in a dose-dependent manner. These results indicate that honokiol has the potential to cause pharmacokinetic drug interactions with other co-administered drugs metabolized by CYP1A2, CYP2C8, CYP2C9, CYP2C19, and UGT1A9. However, it is important to note that the inhibition of CYP activities *in vitro* does not necessarily translate into drug interactions in clinical situations. Thus, clinical trials to evaluate the inhibitory effects of honokiol on CYP1A2, CYP2C8, CYP2C9, CYP2C19, and UGT1A9 should be conducted.
